# The Effects of Major Depressive Disorder on the Sequential Organization of Information Processing Stages: An Event-Related Potential Study

**DOI:** 10.3390/brainsci10120935

**Published:** 2020-12-04

**Authors:** Daniel Kwasi Ahorsu, Ken Chung, Ho Hon Wong, Michael Gar Chung Yiu, Yat Fung Mok, Ka Shun Lei, Hector Wing Hong Tsang

**Affiliations:** 1 Neuropsychiatric Rehabilitation Laboratory, Department of Rehabilitation Sciences, The Hong Kong Polytechnic University, Hung Hom, Hong Kong SAR, China; daniel.ahorsu@connect.polyu.hk (D.K.A.); ken.hm.chung@polyu.edu.hk (K.C.); yatfungmok@gmail.com (Y.F.M.); kslei@polyu.edu.hk (K.S.L.); 2 Yung Fung Shee Psychiatric Center, Department of Psychiatry of United Christian Hospital, The Hong Kong Hospital Authority, Hong Kong SAR, China; whh716@ha.org.hk (H.H.W.); yiugc@ha.org.hk (M.G.C.Y.)

**Keywords:** major depressive disorder, information processing, executive function, community function, event-related potential, choice reaction time task, cognitive-energetical linear stage model, parallel model, linear model

## Abstract

The adverse effects of depression on patients’ life have been reported but information about its effects on the sequential organization of the information processing stages remains poorly understood as previous studies focused only on distinct stages. This study adds to existing knowledge by examining the effect of major depressive disorder (MDD) on the sequential organization of information processing, executive and community functioning. Fifty-seven participants with 19 participants each for first episode depression (FMDD), recurrent episodes depression (RMDD), and healthy controls (HCs) participated in this study. They completed assessments on executive and community functioning measures, and choice reaction time task (CRTT) for the event-related potential (ERP) data. Findings revealed no significant between-group difference in executive functioning but participants with depression (FMDD and RMDD) were found to be more depressed, with FMDD participants having worse community functioning skills compared with HCs. There was no significant between-group main effect on behavioral data. ERP data showed significantly less positive-going P3b among RMDD participants compared with HCs. FMDD participants used a different information processing strategy at P1, while HCs and RMDD participants used a different processing strategy at N2b compared with the other group(s), respectively. The results suggest the use of multifaceted assessment to get a holistic view of the health status of people with MDD in order to inform clinicians on the appropriate interventional strategies needed for the patient.

## 1. Introduction

Depression, especially major depressive disorder (MDD), has been reported to affect all facets of a patient’s life, particularly cognitive processes and functions. More specifically, perception, attention, inhibition, executive function, and memory were reported to be compromised [1,2,3,4]. It is also well documented in behavioral studies that MDD patients suffer impaired decision making or response choices during tasks which may be attributed to their inability to perform or psychomotor retardation [5,6]. Psychomotor retardation, a major symptom of depression, is associated with general slowness and poor performance [7] which can be separated from the effect of arousal or sleepiness [8,9,10,11]. Despite these findings, the effect of depression along information processing stages [12,13] remains poorly understood, especially retardation. This information, however, is very useful for the formulation of more targeted and personalized treatment for people with depression.

**Figure 1:**
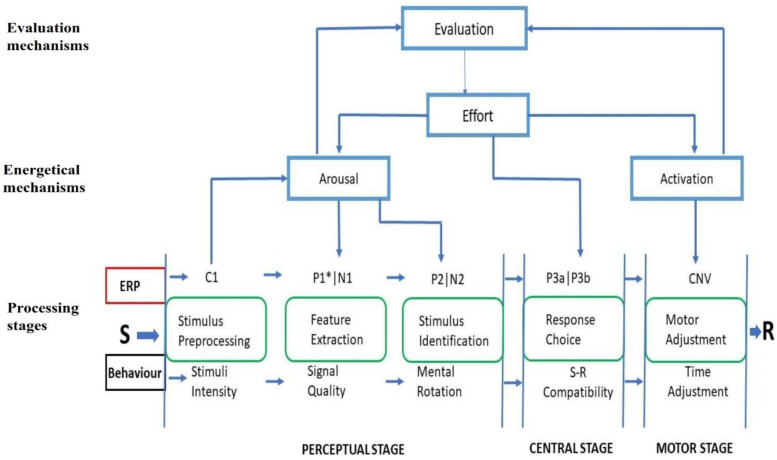
A hypothetical information processing model (h-IPM). This model is adapted from Sanders’ (1983) cognitive-energetical model but it has been modified to include event-related potential (ERP) components (of visual-related tasks) based on several reviews, especially from Luck (2014) [14], Luck and Kappenman (2012) [15], and Woodman (2010) [16]. This proposed model explores and explains why and where there may be challenges in processing information using a novel investigation technique that combines the behavioral and electrophysiological methodologies (perspectives) [14,15,16]. S: Stimulus, R: Response. *P1: Literature review on this component’s exact stage location is vague.

The information processing model (IPM) enables a better understanding of how signals are transformed and transferred through various stages into a final response. Sanders’ cognitive-energetical linear stage model forms the overall theoretical basis of this study (for an extensive review, see Sanders [12,13]). This model has a three-tier mechanism that accommodates the merger between behavioral and electrophysiological (event-related potential; ERP) data (see Figure 1 for the IPM—enhanced). Following this, ERP’s amplitude is linked to the energetical mechanisms of the model, while ERP’s latency is linked to computational processing stages which are time-based. ERP is a well-established method for examining cognition [14,15] and for this reason its data may further enhance our understanding of information processing according to Sanders’ model. Of much more interest to this study is the computational processing stages of the Sanders’ cognitive-energetical linear stage model. Two additional models, the linear and the parallel, are further used to explain the operations of the computational processing stages. The linear (also called discrete or serial) stage model posits that information moves serially from one stage to another with only one stage being active at a time. The parallel (also called overlapping, continuous, or cascade) stage model, in contrary, postulates there could be parallel processing of information among the stages inferred from an interaction between the manipulated variables of the stages (see Sanders [12,13]).

A common factor that underlies these two information processing models (i.e., the linear and the parallel) is time (e.g., reaction time and latency). The models use defined task characteristics that modify the time needed to execute the elementary operation of each stage. The stability and functionality of each information processing stage can, therefore, be tested by manipulating certain variables that are believed to affect a particular stage [13]. Furthermore, manipulation of several stages can be performed to test the type of information processing strategy being used. Hence, no significant interaction between the stages depicts an addictive effect of the stages (linear model), implying the use of the linear processing strategy. A significant interaction between two or more stages, on the other hand, depicts a parallel processing strategy, implying the use of the cascade/overlapping model. Although reaction time (RT), which implicitly reflects the demands on all the stages, can be measured using these manipulations, the qualitative changes in the processing of “capacity” or “resource” part of the information processing model [12,13] are left out. The “capacity” or “resource” part of the information processing model has been proposed to emphasize strategic allocation of “attentional or energetical” resources toward the various mental functions [12,13,17]. This omission can be attributed to the nature of the investigation techniques used which produced behavioral data only. With the emergence of ERP, this gap can be filled using ERP’s amplitudes. By manipulating the stages of information processing, we can therefore explore the effect of MDD on information processing and the sequential organization of its stages.

Previous studies have used choice reaction time task (CRTT) to examine the sequential organization of information processing using behavioral outcomes [5,6,18] as CRTT is usually stable and able to elicit/reveal appropriate responses from stages manipulated [5,6,18]. This study intends to extend the previous findings by adding ERP components. ERP has the obvious advantages in providing information on how information is processed with millisecond precision [19]. Different ERP components relate to different information processes [20], but this study specifically focuses on P1, N1, N2b (as a subcomponent of the N2), P3a, P3b (P3a and P3b as subcomponents of the P3), and contingent negative variation (CNV) components. Using visual-related tasks in ERP studies, components P1 and N1 have been reported to be loosely related to early perceptual processes [1,21] which may represent the feature extraction stage of Sanders’ model [12,13]. Apart from being part of early perceptual processes, the N1 component is also reportedly involved with expert recognition or visual discrimination [16,22]. Some studies have reported that depression does not affect this stage [5,6], while other studies have reported the contrary [17]. The P2 and N2 are loosely related to visual search, stimulus identification, and distinction [20]. However, in relation to the current study, N2b (and sometimes N2b-P3a) is considered to reflect orientating components and it indicates an element of surprise or interest [17,23]. MDD participants with psychomotor retardation were reported to have less N2b-P3a component than HCs [17]. The P3a and P3b reflect automatic processes and controlled processes respectively and both are directly influenced by the effortful phase, especially P3b [12,24]. The response choice/selection stage (P3a and/or P3b) of people with depression has been reported to be affected [6,14,17,25]. Contingent negative variation (CNV) reflects motor preparation or stimulus expectancy during visual-related tasks. It constitutes the activation phase of the energetical mechanisms [12,17,20]. Depression affects CNV, which is also known as motor adjustment in behavioral studies [6]. In addition, previous studies have reported that recurrent episodes MDD (RMDD) patients seem to process information differently than first episode (FMDD) patients which warrants further examination [1,21]. A previous IPM study that used ERP outcomes revealed that depressive patients with retarded-blunted and affect symptoms have slow stimulus encoding (P1) and impaired decision (P3b), while depressive patients with anxious-agitated and impulsive symptoms had quite the opposite results [17]. Another previous ERP study that assessed whether impaired pre-attentive information processing can lead to an impairment of subsequent orienting process revealed that recurrent episodes of depression may lead to impaired pre-attentive information processing, which may cause an impairment of subsequent orienting process as neurophysiological transmission from MMN to P3a [26]. These studies form the background for us to further examine how RMDD and FMDD outpatients compared with HCs differ from processing information from perception to motor preparation (or adjustment).

The response choice (using stimulus-response compatibility) and motor adjustment (using inter-stimulus interval) stages of information processing [13] were specifically chosen for this study due to the stability of the experimental manipulation of variables of these stages [5,17]. Additionally, treating these reported components (RT, P1, N1, N2b, P3a, P3b, and CNV) as outcome measures, we could track the effect of MDD along the information processing stages. Hence, we examined how FMDD patients and RMDD patients differ from HCs in terms of processing information using CRTT with both behavioral and ERP data as outcome measures. To fill the knowledge gap, this study aimed to examine the (1) general differences in speed of information processing and the ERP’s amplitude allocated to these processes depending on particularities of our participants; (2) strategies used for organizing information processing stages among each group of participants; and (3) as a supplementary objective, to explore the interrelationships between executive function outcomes, community functioning, and “RT and latency” outcomes among each group of participants. Hence, three main hypotheses were tested between either FMDD or RMDD participants and HCs (not necessarily between FMDD and RMDD participants). Thus, it was hypothesized that (1) either FMDD or RMDD participants would have significantly different general speed of information processing and/or ERP’s amplitude allocated to these processes compared with HCs; (2) either FMDD or RMDD participants would have significantly different strategies used for organizing information processing stages compared with HCs; and (3) either FMDD or RMDD participants would have significantly different inter-variable (cognitive function, community function, and RT and ERP latencies) relationships compared with HCs. The results would provide important insights to researchers and clinicians on the processing of information (where and why there are impairments) among MDD outpatients and their challenges on cognitive and community functioning.

## 2. Materials and Methods

### 2.1. Participants

The MDD (both FMDD and RMDD) participants were recruited from Yung Fung Shee Psychiatric Clinic (YFSPC) in Hong Kong, the Special Administrative Region of China. YFSPC was a day community psychiatric clinic which provided consultation to outpatients with mental disorders with MDD participants having mild to moderate levels in terms of severity of depression. Most MDD participants came to receive their medications with further therapy or intervention if needed. In general, they usually spent an hour or less per visit. The MDD outpatients were first diagnosed by psychiatrists and further screened for comorbidity using the Structured Clinical Interview for DSM (SCID; [27]). Inclusion criteria for MDD participants were: (1) no comorbidities of other psychiatric disorders such as bipolar and schizophrenia, (2) no psychosis, (3) no neurological conditions, substance abuse, severe head injuries, hypothyroidism, and severe physical illness, and (4) clinically stable with a Mini-Mental State Examination (MMSE) score of 21 or more [28]. The healthy controls were conveniently sampled from surrounding communities using posters. Apart from the inclusion criteria detailed for the MDD participants, HCs did not have depression diagnosis as verified by SCID. Nineteen participants each for FMDD, RMDD, and HC (with a total of 57) who were matched according to sex and handedness were used in this study after a two-year data collection period. This sample size was based on the suggestion by Thirion et al. [29]. These participants participated in other ERP studies in the Cognitive Neuroscience Laboratory (Department of Rehabilitation Sciences of The Hong Kong Polytechnic University, PolyU Laboratory).

### 2.2. Measures/Instrument

For brevity, the measures were grouped into (1) executive function tests and (2) community function test.

#### 2.2.1. Executive Function

##### Cantonese Version of Mini-Mental State Examination (C-MMSE)

This instrument is a 30-item scale used to screen participants for cognitive impairment or mental state in clinical and research settings, especially for dementia. In addition, it was included in this to ensure that the MDD patients were not having moderate/severe cognitive impairment and that they were mentally stable for the study. Hence, the cut-off point for a participant to be included in the study was 21 and above. It has an acceptable internal reliability of α = 0.86 [28].

##### Trail Making Test A and B (TMT-A & B)

This test was used to examine psychomotor speed (TMT-A) and executive function (TMT-B) of participants. TMT comprised the English Trail Making A and Chinese version of Trail Making B [30]. These tests were chosen to corroborate the experimental results on the speed of information processing and response choice/selection. It has a good external validity with Digit Symbol–Coding subtest of the Wechsler Adult Intelligence Scale [31].

##### Modified Card Sorting Test (MCST)

This test was used to assess executive function (sensitive to frontal lobe defect) among participants. Participants were required to respond to 48 cards according to certain rules (set-shifting) in order to evaluate their ability to display flexibility in the face of changing schedules of reinforcement [32,33]. This test was chosen to corroborate the experimental results on response choice/selection.

#### 2.2.2. Community Function

##### Chinese Version of St. Louis Inventory of Community Living Skills (SLICLS)

This scale was used to assess community functioning skills or functional recovery of participants. It has been reported that this scale is able to validly assess community living skills among psychiatric patients across domains such as personal care/physical, social skills, and intellectual skills using local samples in Hong Kong with a high Cronbach alpha of 0.96 [34]. This test was added to the measures to help relate community function with processing speed apart from helping to examine between-group differences.

##### Chinese Version of Beck Depression Inventory-II (C-BDI-II)

This inventory was used to assess the severity level of depression among participants. It also served as the basis for declaring a participant as clinically/psychologically stable. That is, apart from its general score that indicates the severity of depression, we also focused on the suicide items which enabled us to exclude suicidal participants as their behavior might be difficult to control in the course of the study. It has a reliability (alpha) coefficient of 0.92 [35].

### 2.3. Procedure

**Figure 2:**
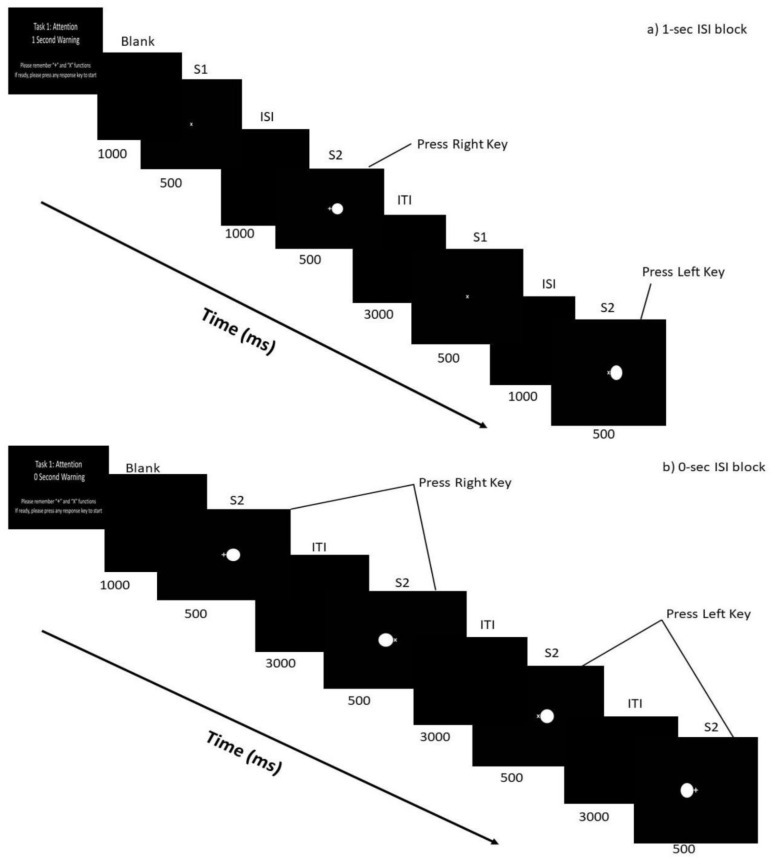
Choice reaction time task (CRTT). This figure gives a detailed stimulus display used in the CRTT according to (**a**) 1-sec Inter-stimulus Interval and (**b**) 0-sec Inter-stimulus Interval block.

A choice reaction-time task (CRTT) with two different visual stimuli was used. A warning stimulus (S1), being a “plus—†” or “cross—X” sign of 1.06 cm^2^ at the center of the monitor which also served as fixation stimuli and an imperative stimulus (S2) which was a circle (2.54 cm in diameter) was attached to either side of the “plus” or “cross” (see Figure 2). The set-up allowed foveal vision of both stimuli. The preparatory period (inter-stimulus interval: ISI) between S1 and S2 was 0 s (in the 0-sec condition, S1 and S2 were presented simultaneously) and 1 s which were presented in different blocks. The participants were explicitly told whether the block was ISI 0 or 1 s. Stimulus-response compatibility (compatible and incompatible) condition was pseudo-randomly distributed within each block of 0-sec or \1-sec ISI. During the compatible (plus—†) condition, the participants responded by pressing the key ipsilateral to the illuminated circle while the incompatible (cross—X) condition required participants to press the key contralateral to the illuminated circle. An equal number of responses for each hand and condition was ensured and the order of condition/situation varied pseudo-randomly within and across tasks for participants. This task was divided into 6 blocks with 48 S1–S2 pairs of trials per block. There were three blocks each for 0-sec ISI and 1-sec ISI. For 1-sec ISI block, S1 was presented for 500 ms, blank screen (with black background) for 1 s, then S2 presented for 500 ms. For 0-sec ISI block, S2 was presented for 500 ms (no S1). Inter-trial interval (ITI) was 3000 ms. There was a minute break after each block and 5 min between the two preparatory period conditions. Participants were fully informed (via an interactive video) on the combinations of both compatibility and ISI conditions followed by practice sessions consisting of a block each for 0-s and 1-sec ISI blocks with compatibility (compatible and incompatible) conditions. Speed and accuracy were stressed for this experiment. Participants were trained on how to focus on the center of the monitor (S1). The entire task lasted approximately 1.5 h. This task was adapted from Pierson, Ragot [17] study. All participants signed the consent form before the assessment and subsequent data collection. Neuropsychological or executive function assessments and electrophysiological recordings were conducted at the PolyU Laboratory. The study was approved by the Research Ethics Committee of the Hong Kong Hospital Authority (KC/KE-16-0114/ER-2) and Research Ethics Committee of The Hong Kong Polytechnic University (PolyU; HSEARS20160523001).

### 2.4. Electrophysiological Recordings

The participants were seated in a dim, sound-proof chamber, approximately 80 cm in front of a 24” DELL LCD monitor, with a high resolution (8.7 pixels/mm’) monitor for stimulus presentation and data acquisition. Participants indicate their choices by pressing the left (1) or right (7) response, key of the Cedrus Stim-tracker response keypad with their corresponding left or right index finger respectively. Electroencephalogram (EEG) was recorded using the 64 channel “Quick-Cap” and referenced to the left mastoid but re-referenced to the link mastoid during offline processing. However, the midline electrodes (e.g., Fz, Cz, Pz) were of interest to this study as recommended by a previous study [17]. The EEG signal was sampled at 1024 Hz. The minimum number of epochs that was permitted for further analyses per ERP component was 216 (75% of stimuli presentation). The CURRY 7 software (NeuroScan Inc., Sterling, VA, USA) was used for signal acquisition as well as offline signal pre-processing of the EEG data. The task was presented using Neuroscan Stim2 software version 4.2 (Neuroscan, El Paso, TX, USA). Electrode impedances were controlled and were always maintained at a value of less than 5 kΩ. Horizontal and vertical ocular movements were simultaneously recorded, and all the data were adjusted for electrooculogram (EOG) artefacts and correction of trials contaminated by blinks and vertical ocular saccades. Signals were low-pass filtered at 30 Hz and high-pass filtered at 0.1 Hz and 24 dB/octave.

### 2.5. Data Analyses

The values of behavioral (accuracy and reaction time—RT) and ERP data (amplitude—Amp and latency—Lat) were based on averaged experimental trials. For the ERP data, the P1 and N1 components were measured at Pz, N2b at Fz, the P3a at Fz, and the P3b at Pz. All ERP latencies were measured using the onset of the stimulus (S2) as reference time. Voltages of ERP peaks were measured with respect to a baseline calculated on the 200 ms preceding the beginning of S2. For the 1-sec ISI, mean amplitudes of CNV were calculated for electrode sites Fz and Cz with respect to a baseline computed on the 200 ms preceding the onset of S1. The time windows 80–170 ms were used for P1, 130–220 ms for N1, 200–270 ms for N2b, 220–360 ms for P3a, 360–550 ms for P3b, and 1300–1500 ms for CNV components based on visual inspection of the grand mean ERP waveforms, a previous study [17] and verified by with independent component analysis (ICA). The ICA, from CURRY 8 (Compumedics Neuroscan, Charlotte, NC, USA), was applied to all channels except EOGs and reference channels. The ICA components with average signal-to-noise ratio (SNR) of 1.0 or greater [36,37] were used to verify the interested components and their corresponding time-windows.

After consultation with our departmental biostatistician and relevant literature [38,39], a three-way mixed factor analysis of variance (ANOVA) was used to assess the statistical significance of between-group differences and intragroup effects of experimental variables. The mixed factor ANOVA consisted of one intergroup factor (Group factor) and two intragroup factors with two modalities for each: stimulus-response compatibility (compatible versus incompatible—Comp factor) and inter-stimulus interval (0-s versus 1-s—ISI factor). Topography of recording sites (Topo) was added as a supplementary intragroup factor for CNV. Right and left responses were analyzed together. For a statistically significant interaction, simple effects with Bonferroni correction were run using the SPSS syntax window. One-way analysis of covariance (ANCOVA, years of education as covariate) and one-way analysis of variance (ANOVA) were used to assess the statistical significance of between-group differences for cognitive and community functioning outcomes, respectively. Independent *t*-test was used to examine between-group differences in depression parameters among only MDD outpatients. Correlation analyses among the groups were conducted only for executive function outcomes, community functioning, and “RT and latency” variables that were significant between the groups (see supplementary Analysis S1). For all tests, the significance level was set at <0.05. A group-based scatterplot analysis was done on reaction time due to its particular importance in IPM studies (see supplementary Analysis S2). To determine differences between the groups, results concerning between-group effects and interaction between groups and intragroup were reported and discussed.

## 3. Results

The results are presented in the five sections, namely the executive function, community function, behavioral data, ERP data, and effect of experimental variables.

### 3.1. Executive Function

There was no significant between-group difference on mental state (MMSE; *F*(2,53) = 1.694, *p* = 0.194), processing speed (TMT-A; *F*(2,53) = 0.513, *p* = 0.601), and executive functioning (TMT-B: *F*(2,53) = 1.176, *p* = 0.317; MCST-Cat: *F*(2,53) = 1.820, *p* = 0.172; MCST-PE: *F*(2,53) = 0.894, *p* = 0.415; MCST-NPE: *F*(2,53) = 1.618, *p* = 0.208; MCST-TE: *F*(2, 53) = 1.698, *p* = 0.193) (see Table 1). 

### 3.2. Community Function

FMDD and RMDD participants had significantly more depression severity scores (*F*(2,54) = 6.472, *p* = 0.003) than HCs (*p* = 0.004 and *p* = 0.023 for the post hoc significance between FMDD and HC and RMDD and HCs, respectively). However, only FMDD outpatients had worst community functioning (*F*(2,54) = 3.721, *p* = 0.031) compared with HCs (*p* = 0.038; see Table 1).

### 3.3. Behavioral Data

There was no significant between-group main effect on accuracy (*F*(2,54) = 2.807, *p* = 0.069, ŋ_p_^2^ = 0.094) or on RT (*F*(2,54) = 1.707, *p* = 0.191, ŋ_p_^2^ = 0.059). However, a significant interaction effect between group and ISI (GroupXISI) was found for accuracy (*F*(2,54) = 3.904, *p* = 0.026, ŋ_p_^2^ = 0.126) but not for RT (*F*(2,54) = 3.166, *p* = 0.050, ŋ_p_^2^ = 0.105). For interaction effect on accuracy, simple effect results showed that HCs (69.76 ± 1.64) had more accurate scores than RMDD (63.18 ± 1.64) participants during 0-sec ISI condition (*p* = 0.019). Additionally, FMDD participants had more accurate scores for 0-sec ISI (66.34 ± 1.64) than 1-sec ISI (62.92 ± 1.99) condition (*p* = 0.027).

### 3.4. ERP Data

#### 3.4.1. P1 Component

**Figure 3:**
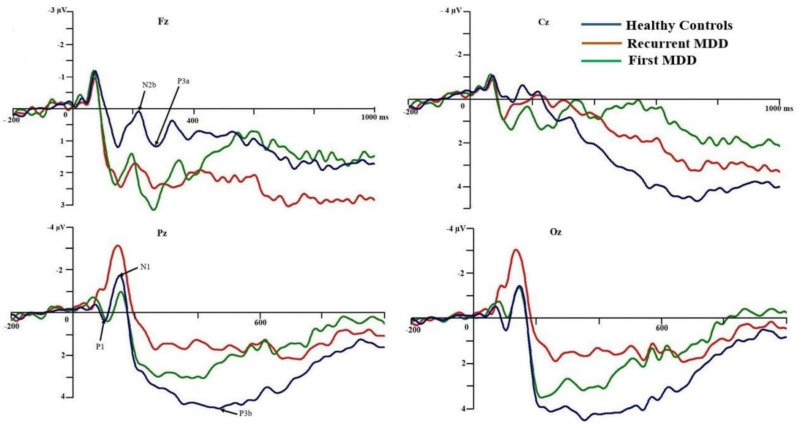
Grand ERP mean waveforms. Grand ERP mean waveform after imperative stimuli (S2) was recorded from the midline electrodes in each group of participants. A reduction of amplitude occurred among RMDD participants (compared with FMDD) at P1 and among RMDD participants (compared with HCs) at P3b.

**Figure 4:**
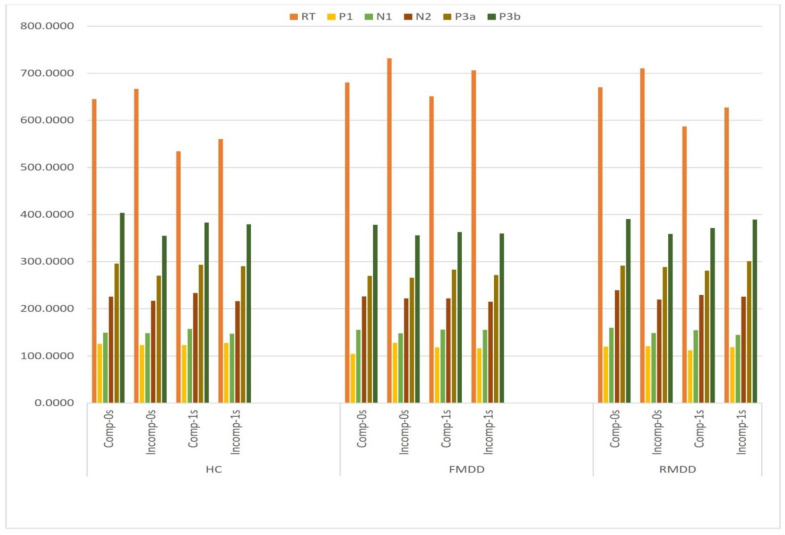
Graphical display of “Reaction Time and ERP latency” results of all the condition according to the group of participants.

A significant main effect of group on P1 amplitude (*F*(2,54) = 3.323, *p* = 0.044, ŋ_p_^2^ = 0.110) was found, with FMDD participants (−0.27 ± 0.54) having a more positive-going amplitude compared with RMDD participants (−2.14 ± 0.54), marginally (*p* = 0.053). However, there was no significant main effect of group on latency (*F*(2,54) = 0.447, *p* = 0.642, ŋ_p_^2^ = 0.016). The Group factor significantly interacted with both intragroup (Comp and ISI) factors on latency (*F*(2,54) = 3.651, *p* = 0.033, ŋ_p_^2^ = 0.119). The interaction effect significantly occurred among FMDD participants only. There was a longer latency with incompatible (118.16 ± 7.59) compared to compatible condition (104.32 ± 7.41) during 0-sec ISI condition (*p* = 0.007). There was also a longer latency during 1-sec ISI (127.55 ± 8.97) than 0-sec ISI (104.32 ± 7.41) during the compatible condition (*p* = 0.006). Further details are presented below in the section examining effects of experimental variables (See Table 2 and Figure 3 and Figure 4). 

#### 3.4.2. N1 Component

There was no significant main effect of group on either amplitude (*F*(2,54) = 2.449, *p* = 0.096, ŋ_p_^2^ = 0.083) or latency (*F*(2,54) = 0.131, *p* = 0.877, ŋ_p_^2^ = 0.005). The other interaction differences were highly insignificant (See Table 2 and Figure 3 and Figure 4).

#### 3.4.3. N2b Component

There was no significant main effect of group on N2b component, either for amplitude (*F*(2,54) = 1.625, *p* = 0.206, ŋ_p_^2^ = 0.057) or latency (*F*(2,54) = 0.672, *p* = 0.515, ŋ_p_^2^ = 0.024). However, the Group factor significantly interacted with both intragroup (Comp and ISI) factors on latency (*F*(2,54) = 3.426, *p* = 0.040, ŋ_p_^2^ = 0.113). The significant interaction effects for N2b latency were among RMDD participants and HCs. RMDD participants had longer latency for compatible (239.53 ± 6.12) than incompatible (229.26 ± 6.28) condition during 0-sec ISI condition (*p* = 0.043). Additionally, RMDD participants had longer latency for 0-sec ISI (239.53 ± 6.12) than 1-sec ISI (219.47 ± 5.63) condition during the compatible condition (*p* = 0.002). HCs also had longer latency for 0-sec ISI (233.26 ± 6.28) than 1-sec ISI (216.11 ± 5.11) condition during the incompatible condition (*p* = 0.006). No other significant interaction effects were observed (all *p*s > 0.05). Further details are presented below in the section examining effects of experimental variables (See Table 2 and Figure 3 and Figure 4).

#### 3.4.4. P3a Component

No significant main effect of group on P3a component, either for amplitude (*F*(2,54) = 1.220, *p* = 0.303, ŋ_p_^2^ = 0.043) or latency (*F*(2,54) = 1.426, *p* = 0.249, ŋ_p_^2^ = 0.050) was found and there was no other significant interaction effect between Group and either Comp and/or ISI factor (all *p*s > 0.05; See Figure 3 and Figure 4).

#### 3.4.5. P3b Component

There was a significant main effect of group on P3b amplitude [*F*(2,54) = 3.191, *p* = 0.049, ŋ_p_^2^ = 0.106] but not its latency (*F*(2,54) = 0.505, *p* = 0.606, ŋ_p_^2^ = 0.018). Bonferroni post hoc comparison revealed that HCs (4.32 ± 0.78) had a more positive-going P3b amplitude than RMDD outpatients (1.57 ± 0.78) (*p* = 0.048). Moreover, the Group factor significantly interacted with ISI factor on latency only (*F*(2,54) = 6.383, *p* = 0.003, ŋ_p_^2^ = 0.191), with HCs (463.84 ± 0.13.29 ms) having a longer latency than FMDD (413.03 ± 13.29 ms) during 0-sec ISI condition (*p* = 0.027). Among HCs, there was a longer latency during 0-sec ISI condition (463.84 ± 0.13.29 ms) than during 1-sec ISI condition (427.26 ± 14.96) (*p* = 0.026). But among FMDD outpatients, there was a shorter latency during 0-sec ISI condition (413.03 ± 13.29 ms) than during 1-sec ISI condition (454.18 ± 14.96 ms) (*p* = 0.013). No other significant interaction effects were observed (all *p*s > 0.05; See Table 2 and Figure 3 and Figure 4).

#### 3.4.6. Post-P3b Latency (RT–P3b Lat)

There was no significant between-group effect on Post-P3b latency (*F*(2,65) = 2.024, *p* = 0.142, ŋ_p_^2^ = 0.070) and there was no significant interaction effect between Group and either Comp and/or ISI factor (all *p*s > 0.05; See Table 2 and Figure 4).

#### 3.4.7. Contingent Negative Variation (CNV)

**Figure 5:**
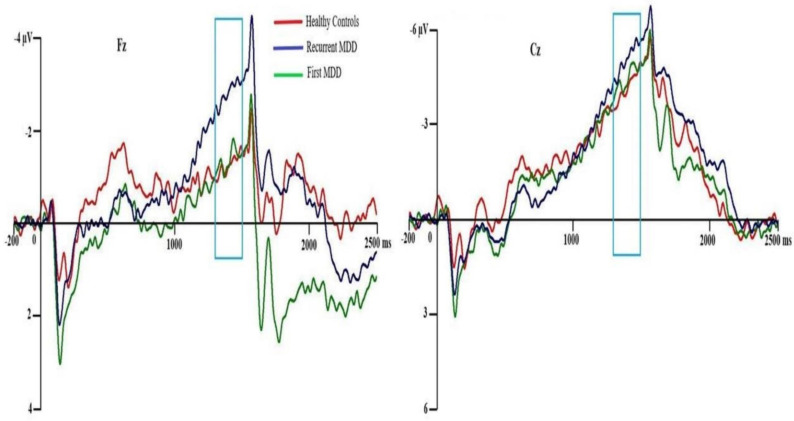
Grand CNV mean waveforms.

There was no significant main effect of group on CNV amplitude (*F*(2,54) = 0.995, *p* = 0.376, ŋ_p_^2^ = 0.036) as shown in Figure 5. However, there was a significant interaction effect between intergroup (Group factor) and intragroup factors (Comp and Topo factors) on CNV amplitude (*F*(2,54) = 4.000, *p* = 0.024, ŋ_p_^2^ = 0.129) (see supplementary Analysis S3 for the full results). No other significant interaction effects were observed (all *p*s > 0.05).

These are grand CNV mean waveforms between the warning (S1) and imperative (S2) stimuli of the midline electrode sites Fz and Cz between all the participants. From the statistical analysis, there was no main effect difference between the groups, although recurrent episode (RMDD) outpatients seem to have a more negative-going CNV amplitude at site Fz than FMDD outpatients and HCs.

## 4. Discussion

In relation to the general between-group differences in speed of information processing and the ERP’s amplitude, the study’s results revealed that there was a significant between-group difference on P3b amplitude. Specifically, HCs had more positive-going P3b amplitude than RMDD outpatients, indicating impaired controlled and effortful processes (response choice) among RMDD outpatients compared with HCs. This main effect was supported by other significant interaction effects. For instance, HCs had more positive-going P3b amplitude than RMDD outpatients during 0-sec ISI condition, suggesting that the impaired controlled and effortful processes (response choice) among RMDD outpatients was when there was no preparatory period. The effect was evidenced by the significantly more accurate scores for HCs than RMDD outpatients during no preparatory period condition (0-sec ISI). This suggests that the RMDD outpatients are likely to have challenges when they are not given ample time to make decisions. On the other hand, FMDD outpatients had significantly shorter P3b latency than HCs during 0-sec ISI. This indicates that FMDD outpatients were faster in making decisions or response choices when there was no time to think about the possible responses or choices. In addition, FMDD outpatients had shorter P3b latency during 0-sec ISI than 1-sec ISI conditions which re-affirms the assertion that they are faster when there was no time to think about the possible responses or choices than when there was. This is contrary to the findings among HCs who had shorter P3b latency during 1-sec ISI than 0-sec ISI conditions. This suggests that FMDD outpatients may have developed compensatory processes which made them perform faster even when there was no preparatory period (i.e., making responses or decision at late processing stage). A previous IPM study that used depressed patients who had “anxious-agitated and impulsive” symptoms reported a shorter P3b latency among them compared with healthy controls [17] which may also be a possibility. The findings among HCs re-affirms the assertion that a preparatory period may reduce the processing time during a task. Specific to this study, HCs benefitted from the effect of the preparatory period but there was a reverse effect among FMDD outpatients. Researchers may further explore the neural mechanisms underlying these observed results using more advanced neuroimaging techniques in association with psychosocial measurement. On the other hand, clinicians may use a preparatory period or cue in guiding patients who have difficulty in making decisions. This main effect is similar to previous studies [6,17] and reviews [2,14,40] which reported that MDD patients had impaired response choice or decision-making process. However, there was no significant between-group (HCs vs MDDs) difference in the main effect for the other exogenous components (P1 and N1) which pertain to early perceptual processes, orienting components (N2b-P3a), automatic processes (P3a), and motor preparation (CNV) which are contrary to previous studies [1,2,5,6,17,25]. These findings (no significant general intergroup differences) imply that MDD outpatients may spend similar processing time and energetical resource (as evidenced by ERP’s amplitude) compared with HCs in perceiving, orienting, and making motor adjustments/preparations. This further indicates that the MDD outpatients, as this study’s findings indicate, have no impaired speed of information processing (RT and ERP latencies) although they have significant challenges with making decisions (specifically P3b amplitude) which partially supports the first hypothesis of this study. Finally, this indicates the need for continuous assessment and rehabilitation until full recovery is achieved among MDD outpatients.

Regarding the organization of information processing stages among the groups (within-group differences), a significant interaction effect between Group and intragroup (ISI and Comp) factors existed among FMDD outpatients at P1 latency but not among RMDD outpatients and HCs. This indicates that FMDD outpatients used the parallel model [12,41] strategy to process information during the early/perceptual information processing stage (P1 latency) so as to save time and make-up for the effect of depression as no significant main effect difference was found for P1 latency. This also suggests that HCs and RMDD outpatients, on the other hand, used the linear model strategy to process information during the early/perceptual information processing stage (P1 latency). This finding is similar to that of a previous study which found that anxious-agitated and impulsively depressed patients used the cascade model at P1 latency [17]. Furthermore, a significant interaction effect between Group and intragroup (ISI and Comp) factors existed among HCs and RMDD outpatients at N2b latency but not among FMDD outpatients, indicating that these groups (HCs and RMDD) used parallel model [12,41] strategy to process information during mid/post-perceptual processing stage in order to achieve a comparatively similar N2b latency main effect while FMDD outpatients used the linear model. This novel finding suggests that RMDD outpatients, like HCs, were flexible in orientational processing of non-emotional stimuli as compared to FMDD outpatients. This flexibility in stimuli orientation processes may be attributed to characteristics such as the duration of depression and recurrence of depressive episodes among RMDD outpatients. These findings shed light on the second aim of the study. Hence, clinicians may need to spend more time to explain the MDD condition at the initial stage and the possible resources available to ease their challenges.

The executive function assessments revealed that there was no significant between-group difference in their mental state and executive functioning. However, MDD outpatients were significantly more depressed than HCs, with both FMDD and RMDD outpatients having a mild level of depression while HCs were within the normal range of the severity scale [35]. In addition, FMDD outpatients had significantly worse community functioning, specifically for social skills, intellectual skills, and in general, community functioning skills compared with HCs. This particular finding is not the resultant effect of only depression as both FMDD and RMDD outpatients had a similar mean score on the depression scale. It can, thus, be suggested that the poorer community function skills among FMDD outpatients stem from the combinatory effects of depression and the first-time experience of the disorder which overwhelms them. The correlational analyses confirmed this. The significant moderate-large [42,43] relationships between community functioning and early to mid-perceptual processes, especially among FMDD outpatients, suggest that their community functioning challenge relates to how they process information at the early to mid-perceptual information processing stages. This possibly accounts for their worse community functioning, especially as no significant relationship was observed among HCs. Hence, they may benefit from social skill and community living skills training. Future studies may explore these assertions. RMDD outpatients, on the other hand, may have gained experience in handling the negative effects of the disorder over time. However, it is surprising that there was no significant between-group difference in cognitive outcomes relative to other studies [44,45,46]. It is assumed that the treatment (medication and therapy) being received partly accounted for this as reported by previous studies [47,48]. Concerning the supplementary objective, the significant interrelationships between the variables among the groups were different suggesting that each group has a different way of processing information and dealing with depression.

This study has some limitations. First, there was no medication-free period for the MDD outpatients although some psychotropic medications affect information processing [45,46]. The absence of a medication-free period in this study was due to the status of MDD participants as outpatients and the study’s compliance with the ethical committee’s requirement. Second, the inclusion-exclusion criteria did put a limit on the sample size as the population size for MDD outpatients without comorbidity also decreased (19 participants, with 16 being females across the groups). The small sample size and the gender disparity, thus, limit the generalization power of the results. Third, researchers should be cautious in over-extending the findings of this study following the small sample size and the multiple data analysis performed.

## 5. Conclusions

In conclusion, the findings demonstrate that behavioral and ERP methods complement each other in revealing the differences in information processing despite the nearly similar cognitive functioning between MDD outpatients and healthy persons. In addition, RMDD outpatients had impaired P3b amplitude, with FMDD outpatients having worse community functioning skills compared with HCs. Comparatively, this study suggests that FMDD outpatients use parallel model during the early/perceptual information processing stage, while RMDD outpatients and HCs use parallel model during mid/post-perceptual processing stage (N2b latency). These findings also provide support why there are between-group differences on the interrelationships among variables. Given these findings, it is imperative that clinicians should, if possible, adopt a multidisciplinary approach for effective biopsychosocial assessment and treatment even at the outpatient phase. Such an approach may offer diverse treatment options (e.g., medical, neuropsychological, brain stimulation, and community intervention) to facilitate recovery among MDD patients. Thus, in as much as feedback or progress report may help guide follow-up treatment approaches, clinicians may request for assessments from other clinicians from other disciplines. Future studies may explore how depression modulate the sequential organization of information processing stages during the processing of emotional stimuli.

## Figures and Tables

**Table 1:** Demographics characteristics of participants.

	FMDD (*n* = 19; 3M ^a^)	RMDD (*n* = 19; 3M ^a^)	HC (*n* = 19; 3M ^a^)	Statistics (ANOVA/ANCOVA)			
	Mean ± SE	Mean ± SE	Mean ± SE	*df*	*t/*F	*p*	Bonferroni Post hocs
Onset Age (years)	33.84 ± 1.97	31.53 ± 1.93	―	36	0.841 ^b^	0.406	―
Illness duration (years)	6.05 ± 1.15	12.42 ± 1.37	―	36	3.557 ^b^	0.001	RMDD > FMDD
Current episode duration (years)	6.05 ± 1.15	3.68 ± 0.85	―	36	1.658 ^b^	0.106	―
No of episode	1 ± 0	2.26 ± 0.17	―	36	7.507 ^b^	0.000	RMDD > FMDD
Age (years)	40.11 ± 8.59	44.47 ± 7.48	38.11 ± 9.28	2	2.802 ^c^	0.070	―
Education (years)	12.11 ± 3.18	11.53 ± 3.50	16.06 ± 1.96	2	13.204 ^c^	0.000	HC > FMDD ***, RMDD ***
MMSE	27.94 ± 0.39	28.92 ± 0.40	28.51 ± 0.43	2	1.694 ^d^	0.194	―
BDI-II	16.37 ± 3.06	14.37 ± 2.24	5.26 ± 1.37	2	6.472 ^c^	0.003	HC < FMDD **, RMDD *
TMT-A	31.53 ± 2.48	32.56 ± 2.56	35.48 ± 2.78	2	0.513 ^d^	0.601	―
TMT-B	57.16 ± 4.15	48.57 ± 4.28	51.18 ± 4.66	2	1.176 ^d^	0.317	―
MCST-Cat	5.37 ± 0.22	5.43 ± 0.22	5.99 ± 0.24	2	1.820 ^d^	0.172	―
MCST-PE	1.77 ± 0.52	1.29 ± 0.54	0.68 ± 0.58	2	0.894 ^d^	0.415	―
MCST-NPE	7.26 ± 0.98	5.91 ± 1.01	4.52 ± 1.10	2	1.618 ^d^	0.208	―
MCST-TE	9.02 ± 1.31	7.39 ± 1.35	5.22 ± 1.47	2	1.698 ^d^	0.193	―
SLICLS-PC/PS	41.68 ± 1.12	41.42 ± 1.44	44.47 ± 0.91	2	2.065 ^c^	0.137	―
SLICLS-SS	23.16 ± 0.72	23.95 ± 0.84	25.89 ± 0.55	2	3.832 ^c^	0.028	HC > FMDD *
SLICLS-IS	22.53 ± 1.00	23.84 ± 0.82	25.95 ± 0.58	2	4.439 ^c^	0.016	HC > FMDD *
SLICLS-Total	87.37 ± 2.64	89.21 ± 0.270	96.32 ± 1.92	2	3.721 ^c^	0.031	HC > FMDD *

^a^ Sex: 3M is 3 males out of 19 participants (16 females, 16F); ^b^ Independent *t*-test; ^c^ One-way ANOVA; ^d^ One-way ANCOVA. All participants are right-handed. MMSE = Mini-Mental State Examination; BDI-II: Becks Depression Inventory-II; SLICLS-PC/PS = St. Louis Inventory of Community Living Skills-Personal Care/Physical Skill; SLICLS-SS = St. Louis Inventory of Community Living Skills-Social Skill; SLICLS-IS = St. Louis Inventory of Community Living Skills-Intellectual Skill; SLICLS = St. Louis Inventory of Community Living Skills. TMT-A = Trail Making Test A; TMT-B = Trail Making Test B; MCST-Cat = Modified Card Sorting Test-Categories; MCST-PE = Modified Card Sorting Test-Perseverative Errors; MCST-NPE = Modified Card Sorting Test-Non-Perseverative Errors; MCST-TE = Modified Card Sorting Test-Total Errors. A < B: A is less than B; A > B: A is greater than B.* *p* < 0.05; ** *p* < 0.01; *** *p* < 0.001.

**Table 2:** Summarized list of “Reaction Time (RT) and latency” commonly ^a^ or differentially ^b^ affected by Inter-stimulus Interval and compatibility in the three groups of participants.

Experimental Variables	Common Effects (for All Groups) ^a^	Differential Effects (Interaction with Group) ^b^
Inter-stimulus interval (ISI)	RT (0-sec > 1-sec)	P3b (0-sec: HC > FMDD), (HC: 0-sec > 1-sec),
	N1 (0-sec > 1-sec)	(FMDD: 1-sec > 0-sec)
	N2b (0-sec > 1-sec)	
	Post-P3b (0-sec > 1-sec)	
Compatibility	RT (Incomp > Comp)	N1 (RMDD: Comp > Incomp)
	Post-P3b (Incomp > Comp)	
ISI X Compatibility	P3b (Comp: 0-sec > 1-sec, Incomp: 1-sec > 0-sec), (0-sec: Comp > Incomp, 1-sec: Incomp > Comp)	P1 (FMDD: Incomp X 0-sec ISI > Comp X 0-sec ISI, 1-sec ISI X Comp > 0-sec ISI X Comp)
	Post-P3b (Comp: 0-sec > 1-sec, Incomp: 0-sec > 1-sec), (0-sec: Inomp > Comp, 1-sec: Incomp > Comp)	N2b (RMDD: Comp X 0-sec ISI > Incomp X 0-sec ISI, 0-sec ISI X Comp > 1-sec ISI X Comp) (HC: 0-sec ISI X Incomp > 1-sec ISI X Incomp)

^a^ Components (e.g., RT, N1 latency) here (common effects) indicate how the groups (FMDD and RMDD, and HCs) are commonly affected by the experimental variables. ^b^ Components (N1, P1, and N2b latency) here (differential effects) indicate how the groups (FMDD and RMDD, and HCs) are differently affected by the experimental variables. A > B: A is longer than B; Comp is compatible condition; Incomp is incompatible condition; 0-sec is 0-sec ISI; 1-sec is 1-sec ISI.

## Supplementary Materials

The following are available online at https://www.mdpi.com/2076-3425/10/12/935/s1, Analysis S1. Correlation between the Executive Functioning, Community Functioning, and Latencies; Analysis S2: Group-based Scatterplot; Analysis S3. Contingent Negative Variation (CNV).
