# Supplementary material for: The Effects of Major Depressive Disorder on the Sequential Organization of Information Processing Stages: An Event-Related Potential Study
Source: Brain Sci. 2020 Dec 4;10(12):935. doi: 10.3390/brainsci10120935 (PMC7761893; doi:10.3390/brainsci10120935)
Supplement: Supplementary file 1 [file brainsci-10-00935-s001.pdf]

Supplementary Analyses

S1. Correlation between the Executive Functioning, Community Functioning, and Latencies

Correlation analyses were conducted for each group using the variables of depression (BDI-II), community functioning (SLICLS), P1, N1, and N2b latencies since there were significant between-group differences. Among HCs, significant moderate-large positive relationships existed between depression and N1 Comp X ISI latency (*p*s < 0.05) (see Table S1). Among FMDD participants, significant moderate-large negative relationships existed between community functioning and depression, N1 compatible X 1-sec ISI latency, and N2b Comp X 0-sec ISI latency (*p*s < 0.05). Also, there were significant moderate-large positive relationships also existed between community functioning N1 compatible X 0-sec ISI latency (*p*s < 0.05) (see Table S2). Among RMDD participants, significant moderate-large positive relationships existed between depression and P1 incompatible X 0-sec ISI latency, N1 compatible-1-sec ISI latency, and N2b Comp X 0-sec ISI latency (*p*s < 0.05). Also, significant moderate-large negative relationships existed between community functioning and N2b compatible X 0-sec ISI latency (*p*s < 0.05) (see Table S3). Other results were not reported as they were intra-scale correlations or outside the aim of the correlational analysis. Also, researchers should be careful in overextending these correlational findings due to the negative effects of multiple testing.

Table S1

Correlation Matrix of variables among Healthy Controls (HCs)

		1	2	3	4	5	6	7	8	9	10	11	12	13	14	15	16	17	18	19	20	21
1	BDI	—																				
2	SLICLS-PC/PS	-0.146	—																			
3	SLICLS-SS	-0.423	0.801**	—																		
4	SLICLS-IS	-0.414	0.846**	0.877**	—																	
5	SLICLS-Total	-0.314	0.955**	0.927**	0.950**	—																
6	P1 Comp-0sec Lat	-0.203	-0.366	-0.198	-0.264	-0.309	—															
7	P1 Comp-1sec Lat	-0.174	-0.313	-0.123	-0.198	-0.243	0.644**	—														
8	P1 Incomp-0sec Lat	-0.187	-0.445	-0.249	-0.309	-0.374	0.858**	0.579**	—													
9	P1 Incomp-1sec Lat	-0.214	-0.253	-0.052	-0.117	-0.170	0.610**	0.928**	0.556*	—												
10	N1 Comp-0sec Lat	0.299	0.035	-0.138	-0.070	-0.044	-0.393	-0.234	-0.309	-0.005	—											
11	N1 Comp-1sec Lat	0.471*	-0.129	-0.282	-0.229	-0.210	-0.170	-0.177	-0.053	-0.067	0.612**	—										
12	N1 Incomp-0sec Lat	0.507*	-0.184	-0.323	-0.262	-0.257	-0.218	-0.241	-0.137	-0.013	0.819**	0.759**	—									
13	N1 Incomp-1sec Lat	0.150	-0.245	-0.453	-0.267	-0.325	-0.102	-0.279	-0.143	-0.340	0.124	0.502*	0.297	—								
14	N2b Comp-0sec Lat	-0.048	-0.277	0.079	-0.098	-0.138	-0.123	-0.043	-0.159	-0.010	0.237	0.185	0.261	0.059	—							
15	N2b Comp-1sec Lat	0.111	-0.118	0.061	-0.149	-0.083	-0.232	-0.263	-0.172	-0.276	0.366	0.370	0.280	0.140	0.825**	—						
16	N2b Incomp-0sec Lat	0.039	-0.314	-0.060	-0.335	-0.267	0.037	0.280	0.095	0.349	0.154	0.255	0.198	-0.151	0.433	0.324	—					
17	N2b Incomp-1sec Lat	0.231	-0.245	-0.016	-0.281	-0.205	-0.086	0.201	0.045	0.204	0.267	0.501*	0.417	0.042	0.605**	0.619**	0.633**	—				
18	P3b Comp-0sec Lat	0.007	-0.309	-0.173	-0.134	-0.236	-0.128	-0.059	-0.065	0.048	0.323	-0.127	0.293	0.087	0.327	0.119	-0.162	0.016	—			
19	P3b Comp-1sec Lat	0.259	-0.244	-0.395	-0.372	-0.339	0.002	-0.031	-0.033	-0.029	0.305	0.535*	0.524*	0.741***	0.124	0.154	-0.026	0.333	0.274	—		
20	P3b Incomp-0sec Lat	-0.178	-0.036	0.230	0.206	0.111	-0.316	-0.268	-0.124	-0.266	-0.128	0.078	-0.042	0.212	0.244	0.152	-0.172	0.155	0.435	0.195	—	
21	P3b Incomp-1sec Lat	-0.153	-0.327	-0.214	-0.117	-0.251	-0.241	-0.140	-0.159	-0.096	0.254	-0.002	0.249	0.263	0.336	0.107	-0.064	0.043	0.711**	0.416	0.510*	—

*p < .05, **p < .01, ***p < .001; The significant correlations in italics are relationships within the same scale and were, therefore, not used in the study. BDI-II: Becks Depression Inventory-II; SLICLS-PC/PS = St. Louis Inventory of Community Living Skills-Personal Care/Physical Skill; SLICLS-SS = St. Louis Inventory of Community Living Skills-Social Skill; SLICLS-IS = St. Louis Inventory of Community Living Skills-Intellectual Skill; SLICLS-Total = St. Louis Inventory of Community Living Skills-Total score.

Table S2

Correlation Matrix of variables among First episode Major Depressive Disorder (FMDD) outpatients

		1	2	3	4	5	6	7	8	9	10	11	12	13	14	15	16	17	18	19	20	21
1	BDI	—																				
2	SLICLS-PC/PS	**-0.564***	—																			
3	SLICLS-SS	**-0.599****	0.758**	—																		
4	SLICLS-IS	**-0.590****	0.869**	0.667**	—																	
5	SLICLS-Total	**-0.630****	0.964**	0.853**	0.933**	—																
6	P1 Comp-0sec Lat	0.060	-0.281	-0.092	-0.363	-0.282	—															
7	P1 Comp-1sec Lat	-0.254	0.016	-0.075	-0.027	-0.025	0.598**	—														
8	P1 Incomp-0sec Lat	0.227	-0.302	-0.320	-0.383	-0.363	0.735**	0.531*	—													
9	P1 Incomp-1sec Lat	-0.306	0.256	0.123	0.281	0.249	*0.469**	*0.712***	0.339	—												
10	N1 Comp-0sec Lat	-0.369	0.367	**0.480***	**0.508***	**0.482***	0.187	-0.082	0.115	0.080	—											
11	N1 Comp-1sec Lat	0.056	-0.328	-0.023	**-0.558***	-0.356	0.373	-0.056	0.113	-0.401	-0.137	—										
12	N1 Incomp-0sec Lat	-0.337	0.271	0.379	0.447	0.390	0.160	-0.066	0.029	0.122	*0.800***	-0.180	—									
13	N1 Incomp-1sec Lat	-0.196	0.053	0.279	-0.066	0.076	**0.510***	0.047	0.313	0.010	*0.568**	0.450	0.466*	—								

		1	2	3	4	5	6	7	8	9	10	11	12	13	14	15	16	17	18	19	20	21
14	N2b Comp-0sec Lat	0.080	-0.164	**-0.516***	-0.193	-0.287	-0.055	0.124	0.136	0.194	-0.445	-0.229	-0.280	-0.373	—							
15	N2b Comp-1sec Lat	0.237	-0.036	-0.058	-0.176	-0.098	0.086	-0.025	0.272	0.079	-0.229	0.002	-0.350	-0.316	0.404	—						
16	N2b Incomp-0sec Lat	0.179	-0.327	**-0.486***	-0.386	-0.421	0.257	0.142	0.396	-0.010	-0.359	0.179	-0.223	-0.264	0.739**	0.506*	—					
17	N2b Incomp-1sec Lat	0.312	-0.086	-0.355	-0.187	-0.207	-0.099	-0.276	0.245	-0.120	-0.279	-0.017	-0.276	-0.167	.530*	0.538*	0.622**	—				
18	P3b Comp-0sec Lat	0.410	0.049	0.089	-0.125	-0.001	0.030	-0.337	-0.063	-0.288	-0.070	0.215	.099	-.114	-.051	0.466*	0.182	0.158	—			
19	P3b Comp-1sec Lat	0.256	-.275	0.013	-0.283	-0.220	0.121	-0.078	-0.163	0.027	-0.314	0.082	-0.296	-0.244	0.149	0.551*	0.224	0.183	0.314	—		
20	P3b Incomp-0sec Lat	0.353	-0.057	0.062	-0.120	-0.052	-0.232	-0.453	-0.289	-0.402	-0.203	0.173	0.191	-0.174	-0.038	0.011	0.157	0.109	0.667**	0.124	—	.
21	P3b Incomp-1sec Lat	-0.169	-0.047	0.222	-0.051	0.023	0.200	0.131	-0.111	0.219	0.065	-0.031	-0.019	0.045	-0.206	0.207	-0.165	-0.152	-0.003	0.655**	-0.301	—

*p < .05, **p < .01; The significant correlations in italics are relationships within the same scale and were, therefore, not used in the study. BDI-II: Becks Depression Inventory-II; SLICLS-PC/PS = St. Louis Inventory of Community Living Skills-Personal Care/Physical Skill; SLICLS-SS = St. Louis Inventory of Community Living Skills-Social Skill; SLICLS-IS = St. Louis Inventory of Community Living Skills-Intellectual Skill; SLICLS-Total = St. Louis Inventory of Community Living Skills-Total score.

Table S3

Correlation Matrix of variables among Recurrent episode Major Depressive Disorder (RMDD) outpatients

		1	2	3	4	5	6	7	8	9	10	11	12	13	14	15	16	17	18	19	20	21
1	BDI	—																				
2	SLICLS-PC/PS	-0.316	—																			
3	SLICLS-SS	-0.238	0.752**	—																		

4	SLICLS-IS	-0.400	0.469*	0.661**	—																
5	SLICLS-Total	-0.364	0.910**	0.913**	0.760**	—															
6	P1 Comp-0sec Lat	0.316	-0.244	-0.161	-0.086	-0.206	—														
7	P1 Comp-1sec Lat	0.103	0.037	0.156	0.093	0.096	0.345	—													
8	P1 Incomp-0sec Lat	**0.533***	-0.349	-0.210	-0.098	-0.281	0.787**	0.400	—												
9	P1 Incomp-1sec Lat	-0.178	-0.360	-0.300	-0.235	-0.357	0.325	0.464*	0.043	—											
10	N1 Comp-0sec Lat	0.000	0.146	0.087	-0.113	0.071	-0.367	-0.209	-0.100	**-0.512***	—										
11	N1 Comp-1sec Lat	**0.468***	-0.036	-0.299	-0.305	-0.205	-0.020	-0.310	0.109	-0.428	0.417	—									
12	N1 Incomp-0sec Lat	0.042	0.189	-0.022	-0.196	0.035	-0.360	-0.305	-0.160	**-0.599****	0.921**	0.550*	—								
13	N1 Incomp-1sec Lat	0.382	-0.023	-0.240	-0.174	-0.139	-0.025	-0.389	0.064	-0.454	0.457*	0.793**	0.599**	—							
14	N2b Comp-0sec Lat	**0.493***	**-0.506***	-0.334	-0.291	**-0.462***	0.120	-0.354	0.278	-0.228	0.078	0.287	0.107	0.394	—						
15	N2b Comp-1sec Lat	-0.043	-0.281	-0.082	0.291	-0.087	0.050	0.206	0.296	-0.071	-0.044	-0.023	-0.103	-0.109	0.312	—					
16	N2b Incomp-0sec Lat	**0.660****	-0.418	-0.335	-0.417	-0.454	0.203	-0.058	0.340	-0.121	0.062	0.316	0.125	0.361	0.846**	0.139	—				
17	N2b Incomp-1sec Lat	-0.080	0.122	0.242	0.258	0.219	-0.002	-0.251	-0.093	-0.230	-0.243	-0.303	-0.197	-0.144	0.501*	0.363	0.241	—			
18	P3b Comp-0sec Lat	0.086	0.146	0.285	**0.581****	0.343	-0.164	0.168	-0.158	-0.173	-0.124	-0.084	-0.149	-0.073	-0.077	0.036	-0.052	0.136	—		
19	P3b Comp-1sec Lat	-0.025	0.441	0.292	0.142	0.369	-0.121	-0.169	-.050	-.222	0.066	0.012	0.090	-0.247	-0.397	-0.332	-0.396	-0.118	0.022	—	.
20	P3b Incomp-0sec Lat	0.338	0.148	0.325	0.081	0.205	-0.132	0.216	-0.116	-0.054	-0.236	-0.052	-0.224	-0.326	-0.164	-0.162	-0.071	-0.010	0.535*	0.323	—

21	P3b Incomp-1sec Lat	0.088	0.299	**0.467***	0.125	0.343	-0.124	-0.021	-0.153	-0.064	-0.172	-0.211	-0.233	-0.412	-0.301	-0.326	-0.313	0.040	0.235	0.650**	0.698**	—

*p < .05, **p < .01; The significant correlations in italics are relationships within the same scale and were, therefore, not used in the study. BDI-II: Becks Depression Inventory-II; SLICLS-PC/PS = St. Louis Inventory of Community Living Skills-Personal Care/Physical Skill; SLICLS-SS = St. Louis Inventory of Community Living Skills-Social Skill; SLICLS-IS = St. Louis Inventory of Community Living Skills-Intellectual Skill; SLICLS-Total = St. Louis Inventory of Community Living Skills-Total score

S2: Group-based Scatterplot

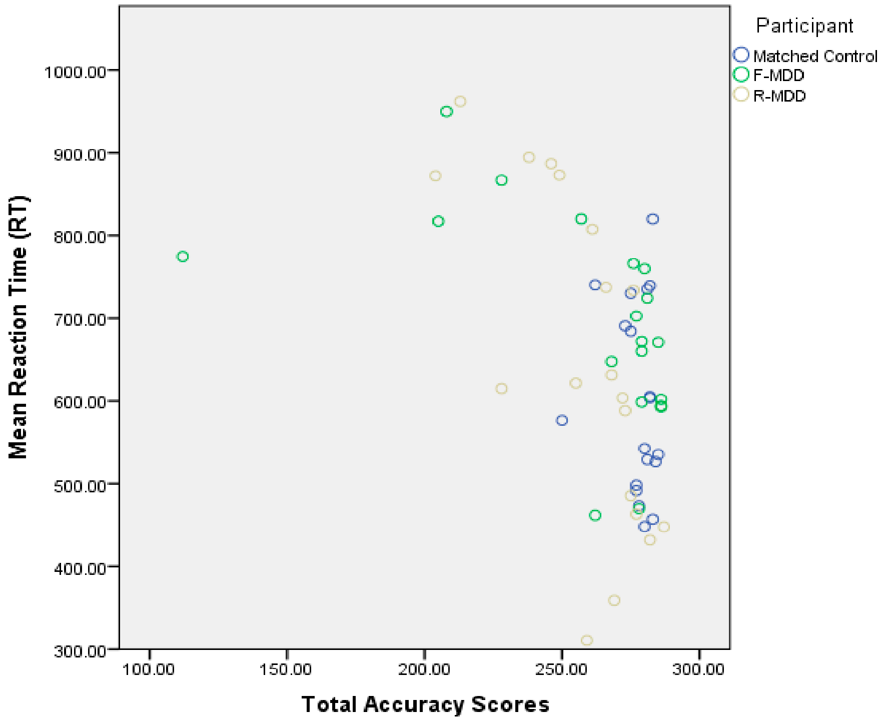

Figure S1: Group-based scatterplot of Accuracy on RT

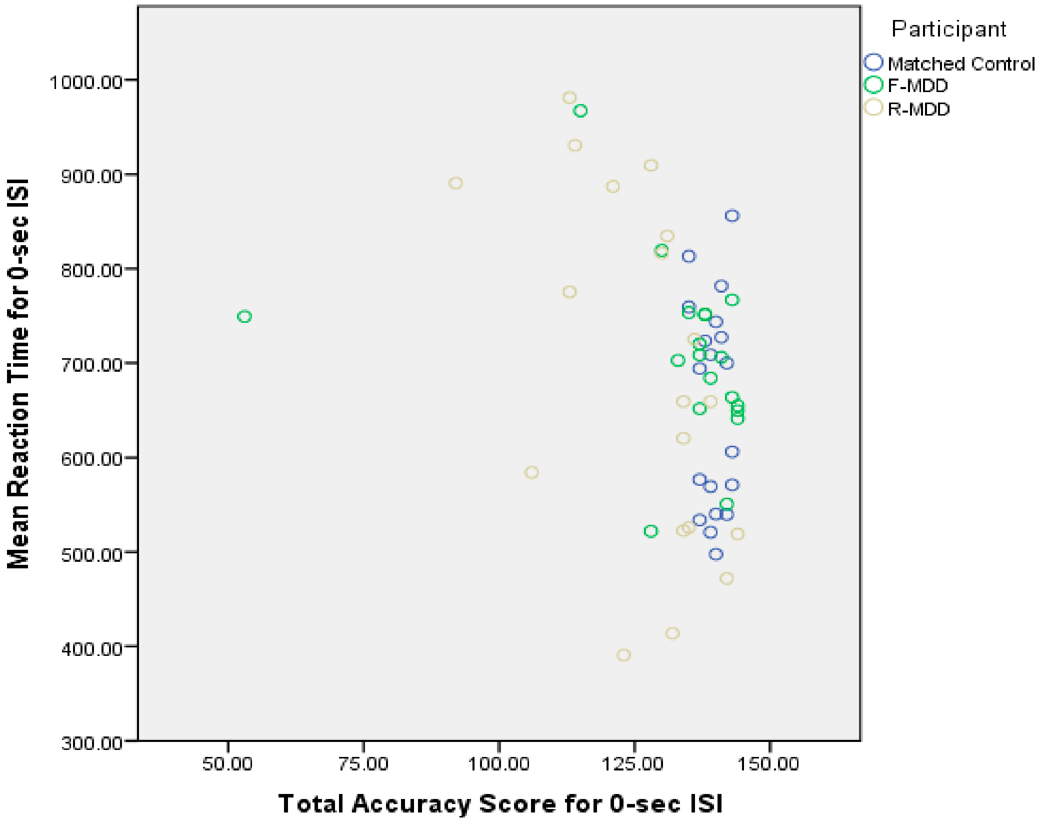

Figure S2: Group-based scatterplot of Accuracy on RT (0-sec ISI)

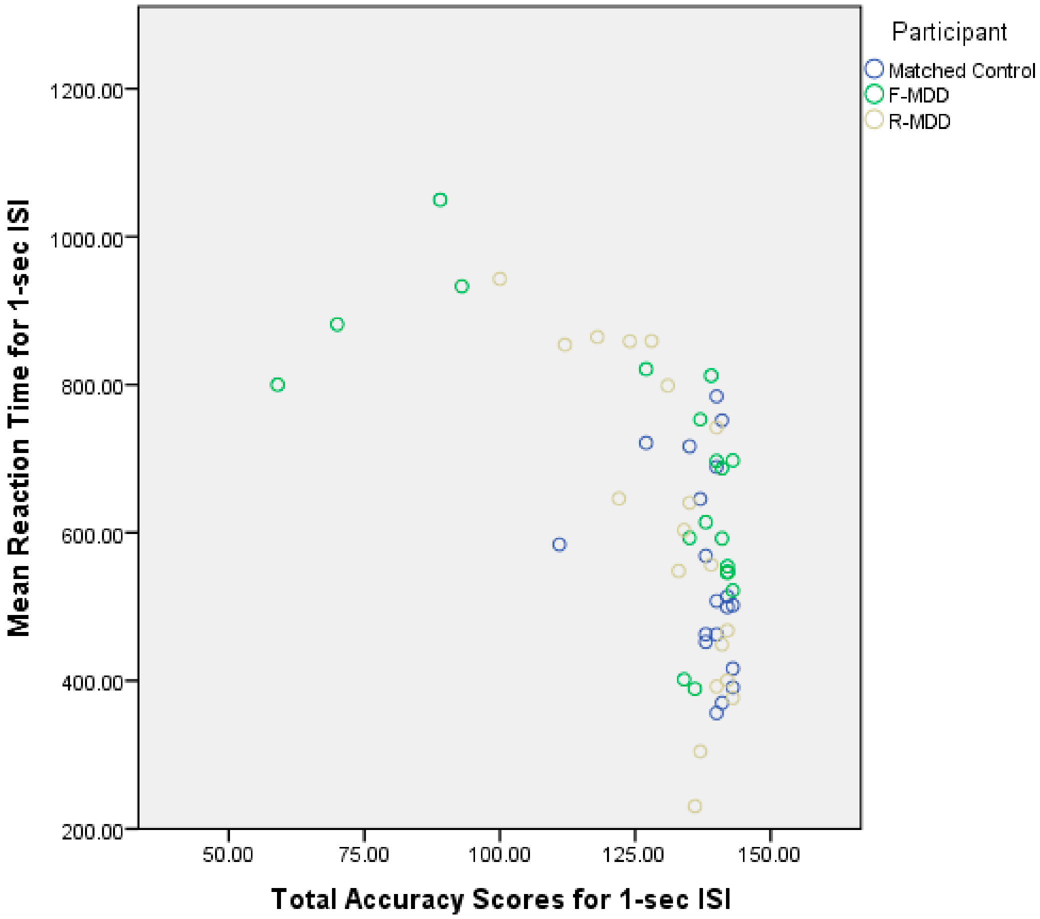

Figure S3: Group-based scatterplot of Accuracy on RT (1-sec ISI)

Figures S1 to S3 showed a data that were mostly clustered around the maximum accuracy score for the task. Figures S1 and S2 had a data that was suggestive of being an outlier. However, there was no significant between-group differences after removing it from the data. Hence, it was maintained in order to help match the other participants (i.e., participants in groups were matched).

S3. Contingent Negative Variation (CNV)

There was a significant interaction effect between intergroup (Group factor) and intragroup factors (Comp and Topo factors) on CNV amplitude ($F(2,54) = 4.000$, $p = .024$, $\eta_p^2 = .129$). Among RMDD outpatients, there was a more negative-going CNV amplitude during incompatible condition (-5.95 ± 1.04 μV) than compatible condition (-4.73 ± 0.94 μV) at site Cz ($p < .024$). Furthermore, among each of the groups, it was observed that there was a more negative-going CNV amplitude at site Cz (HCs (-4.67 ± 0.94 μV), FMDD (-4.02 ± 0.94 μV) and RMDD (-4.73 ± 0.94 μV) outpatients) than site Fz (HCs (-1.59 ± 0.58 μV), FMDD (-1.21 ± 0.58 μV) and RMDD (-2.22 ± 0.58 μV)) during compatible condition (all *ps* = .000) and a more negative-going CNV amplitude at site Cz (HCs (-4.28 ± 1.04 μV), FMDD (-4.44 ± 1.04 μV) and RMDD (-5.95 ± 1.04 μV) outpatients) than site Fz (HCs (-1.52 ± 0.64 μV), FMDD (-0.67 ± 0.64 μV) and RMDD (-3.06 ± 0.64 μV) outpatients) during incompatible condition (all *ps* = 0.000). No other significant interaction effects were observed (all *ps* > .05).
